# Electroconvulsive Therapy Response in Major Depressive Disorder: A Pilot Functional Network Connectivity Resting State fMRI Investigation

**DOI:** 10.3389/fpsyt.2013.00010

**Published:** 2013-03-01

**Authors:** Christopher C. Abbott, Nicholas T. Lemke, Shruti Gopal, Robert J. Thoma, Juan Bustillo, Vince D. Calhoun, Jessica A. Turner

**Affiliations:** ^1^Department of Psychiatry, School of Medicine, University of New MexicoAlbuquerque, NM, USA; ^2^The Mind Research NetworkAlbuquerque, NM, USA; ^3^Chester F. Carlson Center for Imaging Science, Rochester Institute of TechnologyRochester, NY, USA; ^4^Department of Neurosciences, School of Medicine, University of New MexicoAlbuquerque, NM, USA; ^5^Department of Electrical and Computer Engineering, University of New MexicoAlbuquerque, NM, USA

**Keywords:** major depressive disorder, electroconvulsive therapy, resting state fMRI, independent component analysis, functional network connectivity

## Abstract

Major depressive disorder (MDD) is associated with increased functional connectivity in specific neural networks. Electroconvulsive therapy (ECT), the gold-standard treatment for acute, treatment-resistant MDD, but temporal dependencies between networks associated with ECT response have yet to be investigated. In the present longitudinal, case–control investigation, we used independent component analysis to identify distinct networks of brain regions with temporally coherent hemodynamic signal change and functional network connectivity (FNC) to assess component time course correlations across these networks. MDD subjects completed imaging and clinical assessments immediately prior to the ECT series and a minimum of 5 days after the last ECT treatment. We focused our analysis on four networks affected in MDD: the subcallosal cingulate gyrus, default mode, dorsal lateral prefrontal cortex, and dorsal medial prefrontal cortex (DMPFC). In an older sample of ECT subjects (*n* = 12) with MDD, remission associated with the ECT series reverses the relationship from negative to positive between the posterior default mode (p_DM) and two other networks: the DMPFC and left dorsal lateral prefrontal cortex (l_DLPFC). Relative to demographically healthy subjects (*n* = 12), the FNC between the p_DM areas and the DMPFC normalizes with ECT response. The FNC changes following treatment did not correlate with symptom improvement; however, a direct comparison between ECT remitters and non-remitters showed the pattern of increased FNC between the p_DM and l_DLPFC following ECT to be specific to those who responded to the treatment. The differences between ECT remitters and non-remitters suggest that this increased FNC between p_DM areas and the left dorsolateral prefrontal cortex is a neural correlate and potential biomarker of recovery from a depressed episode.

## Introduction

Electroconvulsive therapy (ECT) remains the gold-standard treatment for severe, treatment-resistant patients with major depressive disorder (MDD) where a rapid response is indicated. The ECT success rate in MDD, the most common diagnostic indication for the estimated 100,000 annual ECT treatments in the U.S., is approximately 75% (Hermann et al., 1995; Weiner et al., 2001). During a 3–4 week course of an ECT series, most depressive episodes remit, and formerly suicidal or psychotically depressed patients will resume their premorbid levels of functioning. The short time interval and magnitude of response make ECT an ideal therapeutic intervention to assess biomarkers of response in MDD. Resting state functional magnetic resonance imaging (fMRI) has recently expanded the scope and generalizability of fMRI investigations to include patients with severe MDD treated with ECT (Beall et al., 2012; Perrin et al., 2012).

Functional connectivity in resting fMRI data has become a widely used technique and can be measured in various ways (Erhardt et al., 2011a). The two most widely used approaches include the use of a seed-based method (Biswal et al., 1995) and spatial independent component analysis (ICA; McKeown et al., 1998; Calhoun and Adali, 2012). A cross-sectional seed-based approach of MDD revealed increased temporal coherence within limbic, cortical, and default mode networks (Sheline et al., 2010). Furthermore, these networks overlapped with an area of the dorsal medial prefrontal cortex (DMPFC). The increased temporal coherence of these brain regions may be an important therapeutic target in MDD. Perrin et al. (2012) tested this hypothesis with a longitudinal resting state fMRI investigation and found that ECT response was associated with reduced temporal coherence within the left dorsal lateral prefrontal cortex (l_DLPFC; Perrin et al., 2012).

In contrast to the seed-based approach, spatial ICA utilizes a data-driven multivariate approach to identify distinct groups of brain regions with temporally coherent (and hence functionally connected) hemodynamic signal change (Calhoun et al., 2008). While the ICA spatial maps are maximally independent, their respective time courses can have considerable temporal dependencies. Functional network connectivity (FNC) measures correlations between component time courses (Jafri et al., 2008). FNC has been applied to fMRI investigations of schizophrenia, aging, and neurodegenerative disorders (Jafri et al., 2008; Allen et al., 2011; Filippi et al., 2012). The longitudinal differences in FNC associated with remission from a depressed episode have yet to be investigated.

The pathophysiology of MDD can be conceptualized as a “systems-level” disorder affecting multiple brain areas and their related neurotransmitter systems (Mayberg, 2003; Mayberg et al., 2005). Functionally integrated networks or pathways in cortical and limbic regions that fail to maintain homeostatic emotional control may result in affective, cognitive, and neurovegetative symptoms of depression. In the present investigation, we focus our analysis on four regions (or components) affected in MDD: the subcallosal cingulate gyrus (SCC), default mode network, dorsal lateral prefrontal cortex, and DMPFC (Greicius et al., 2007; Sheline et al., 2010). Previous cross-sectional fMRI studies have shown increased connectivity in these networks in MDD relative to healthy comparison subjects with seed-voxel correlations (Sheline et al., 2010) and ICA (Greicius et al., 2007). Furthermore, a recent resting state fMRI investigation has shown decreased connectivity in the dorsolateral prefrontal cortex in MDD associated with ECT response (Perrin et al., 2012). First, we assessed differences in the longitudinal pre- and post-ECT data. Second, we compared the pre-ECT and post-ECT data with demographically matched healthy comparisons to assess the degree of normalization associated with ECT response. Third, we compared differences in FNC between ECT remitters versus non-remitters. We defined aberrant FNC as differences in the MDD group relative to the healthy comparisons subjects. We hypothesized that ECT response would be associated with normalization of aberrant FNC relationships.

## Materials and Methods

### Participants

Prior to initiating this study, ethical approval was obtained from the Human Research Protections Office at the University of New Mexico (UNM), and the study was conducted in accordance with the principles expressed in the Declaration of Helsinki. Patients were recruited from the UNM Mental Health Center’s inpatient and outpatient services. Patients had decisional capacity or assented with a surrogate decision maker providing formal consent. For this investigation, depressed patients met the following inclusion criteria: (1) DSM-IV TR diagnosis of MDD made be a board-certified geriatric psychiatrist (CA); (2) the clinical indications for ECT including treatment resistance and a need for a rapid and definitive response (Weiner et al., 2001); (3) a Hamilton Depression Rating Scale – 24 item (HDRS-24) ≥ 21 (Kellner et al., 2006); and (4) age ≥50 years to reduce age-related heterogeneity. Exclusionary criteria included the following: (1) defined neurological or neurodegenerative disorder (e.g., head injury or epilepsy, Alzheimer’s disease); (2) other psychiatric conditions (e.g., schizophrenia, schizoaffective disorder, Bipolar I or II disorder); (3) current drug or alcohol dependence; (4) contraindications to MRI (e.g., pacemaker); and (5) pregnancy.

Age- and gender-matched healthy comparison participants were recruited from the same demographic area and completed one session of resting state fMRI using the identical imaging protocol. Additional exclusion criteria for the healthy comparison group included psychiatric diagnosis and current use of psychotropic medications. The use of cross-sectional data for the comparison subjects is consistent with other longitudinal case–control studies assessing treatment effects with resting state data in neuropsychiatric disorders (Lui et al., 2010). Previous resting state studies have shown a high level of consistency in healthy individuals (Harrison et al., 2001; Shehzad et al., 2009; Guo et al., 2012).

### Clinical assessments

Patients receiving ECT underwent clinical assessments with the HDRS-24 and Hamilton Endogenomorphic Scale (HES; Thase et al., 1983) and cognitive assessments with the Repeatable Battery for the Assessment of Neuropsychological Status (RBANS; Randolph et al., 1998) and the Trail Making Test Parts A and B (Reitan, 1958) before and after the ECT series. The initial assessment occurred 1–2 days prior to ECT series, and the final imaging assessment followed the last ECT treatment by a minimum of 5 days. The delay from the last ECT treatment to the post-ECT scan minimized the subacute effects of the seizure (Schmidt et al., 2008). Patients were considered remitters if they had a 60% reduction in pretreatment HDRS-24 and a maximum post-treatment score of 10 following the ECT series (Sackeim et al., 2001).

### Electroconvulsive therapy

The anesthetic agents included methohexital (1 mg/kg) and succinylcholine (1 mg/kg). Clinical judgment from the ECT physician determined lead placement at the start of the ECT series. A Thymatron System IV (Somatics LLC, Lake Bluff, IL, USA) delivered a right unilateral (*n* = 10) or bitemporal ECT (*n* = 2) stimulus delivery with a constant-current, brief pulse (0.50 ms). For the bitemporal stimulus delivery, the center of the stimulus electrodes were placed 3 cm above a line connecting the canthus of the eye and the external auditory meatus (Kellner et al., 2010). For the right unilateral stimulus delivery, the right temporal lead was placed as previously described. Another lead was placed 3 cm lateral to the right of the vertex of the skull. Seizure threshold obtained during the first session with a dose titration method guided subsequent stimulus dosage (6 × threshold for right unilateral, 2 × threshold for bitemporal; Kellner et al., 2010). Treatments occurred thrice weekly (Monday, Wednesday, and Friday) until adequate clinical response or clinical decision to stop treatment in the context of non-response (11.17 ± 3.33 sessions in the series).

### MRI and fMRI data acquisition

All MRI images were collected on the Mind Research Network (MRN) 3-Tesla Siemens Trio scanner. High-resolution T1-weighted structural images were acquired with a 5-echo MPRAGE sequence with TE = (1.64, 3.5, 5.36, 7.22, 9.08) ms, TR = 2.53 s, TI = 1.2 s, flip angle = 7, number of excitations = 1, slice thickness = 1 mm, field of view = 256 mm, resolution = 256 × 256. T2-weighted functional images were acquired with a gradient-echo EPI sequence with TE = 29 ms, TR = 2 s, flip angle = 75, slice thickness = 3.5 mm, slice gap = 1.05 mm, field of view 240 mm, matrix size = 64 × 64, voxel size = 3.75 × 3.75 × 4.55 mm. Resting state scans were acquired over a minimum of 5 min, 16 s in duration (158 volumes). Subjects were instructed to keep their eyes open during the scan and stare passively at a fixation cross.

### Structural and fMRI image processing

An automated pipeline and neuroinformatics system developed at the MRN and based on Statistical Parametric Mapping 5 (SPM5)^1^ preprocessed the functional and structural MRI data (Scott et al., 2011). In the functional data pipeline, the first four volumes were discarded to remove T1 equilibration effects. Images were realigned with INRIalign (Freire et al., 2002), and slice-timing correction was applied with the middle slice as the reference frame. Data were then spatially normalized into the standard Montreal Neurological Institute (MNI) space, resliced to 3 mm × 3 mm × 3 mm voxels and smoothed using a Gaussian kernel with a full-width at half-maximum (FWHM) of 10 mm.

### Independent component analysis

Group ICA (Calhoun et al., 2001) was performed using the Group ICA fMRI Toolbox (GIFT)^2^. In contrast to the seed-based approach, spatial ICA utilizes a data-driven multivariate approach to identify distinct groups of brain regions with temporally coherent (and hence functionally connected) hemodynamic signal change (Calhoun et al., 2008). The advantages of ICA over seed-based correlational techniques include the following: (1) eliminates the arbitrary choice of seed-voxel, (2) takes into account all between voxel information, (3) successfully identifies and removes motion-related sources, and (4) increases sensitivity to detect group differences (McKeown et al., 2003; Kochiyama et al., 2005; Koch et al., 2010; Allen et al., 2011).

The preprocessed fMRI data were reduced in two steps. First, subject-level data dimensionality was reduced to 100. Second, the concatenated, aggregate data was further reduced to 75. The relatively higher model order (Components, *C* = 75) identified components that correspond with known functional networks (Ystad et al., 2010). The Infomax algorithm was repeated 20 times with ICASSO to maximize the reliability and robustness of the component spatial maps. Subject specific time courses and spatial maps were then back reconstructed (Erhardt et al., 2011b). Three raters (Christopher C. Abbott, Shruti Gopal, and Jessica A. Turner) used visual inspection of spatial maps and low frequency power spectra to select the components of interest (Cordes et al., 2000; Allen et al., 2011).

### Functional network connectivity

The FNC Toolbox (FNCtb)^3^ bandpassed filtered the ICA time courses from 0.01 to 0.10 Hz and computed the differences in lagged correlations (±3 s) between pairs of the selected components (6 components, 15 pairs of correlations; Jafri et al., 2008). Fisher’s transformation converted each correlation to a *z*-score prior to the statistical analysis.

### Statistical analysis

Because of the small sample size, we assessed normality assumptions with box plots and Levene’s test for equality of variance on the demographic, clinical, and FNC Fisher transformed data. For the longitudinal differences in symptom scores (HDRS-24 and HES), we used non-parametric statistics (paired-sample Wilcoxon signed-rank test) to assess longitudinal differences before and after the ECT series.

Within ECT remitters, we assessed longitudinal differences (pre- and post-ECT) in FNC with paired *t*-tests. We used a false discovery rate (*P* < 0.05) to correct for multiple comparisons (Genovese et al., 2002).

Following these analyses, we compared pre-ECT FNC measures on the significant pairs of networks to the same measures in healthy subjects using a two-sample *t*-test. We also compared post-ECT measures to the healthy subject FNC measurements in the same way. Significant threshold were set to *P* < 0.05.

We correlated the change in FNC measures with the change in symptom measures for all subjects, and for ECT remitters only with a significance threshold of *P* < 0.05.

Finally, a two-factor analysis of variance assessed longitudinal changes in FNC between group (ECT remitters and non-remitters) and time (pre- and post-ECT). A two-factor analysis of variance also assessed differences in stimulus delivery (bitemporal and right unilateral) and time (pre- and post-ECT).

## Results

### Participants

The average age for the depressed patients (*n* = 12) was 66.42 ± 9.78 years (four male/eight females). Eleven of the twelve depressed subjects started this study during an inpatient psychiatric hospitalization. Three subjects had a depressive episode with psychotic features and the remaining subjects had non-psychotic depressive episodes. Eleven of the twelve depressed subjects had a history of recurrent depressive episodes. All depressed subjects were treated with antidepressant medications throughout this investigation. Eight subjects were concurrently treated with antipsychotics, and two subjects were treated with a mood stabilizer (lamotrigine). Medication changes between the two imaging assessments were minimal and consisted of an antidepressant cross titration (*n* = 1), antidepressant discontinuation (*n* = 2), and the addition of an antipsychotic (olanzapine, *n* = 1). The healthy comparison subjects (*n* = 12) were matched for age and gender (age *t*_22_ = 0.90, *P* = 0.90; gender *x*^2^ = 0.00, *P* = 1.00). The demographic and clinical characteristics of the patients and comparison subjects are shown in Table 1.

**Table 1:** **The top section includes the demographic variables of the subjects with major depressive disorder (MDD) and demographically matched healthy comparison subjects (HC)**. The lower section includes clinical symptom ratings and neuropsychological indices (RBANS index scores, Trail Making Test Parts A and B in seconds).

Demographics	MDD mean (SD) or ratio	HC mean (SD) or ratio	*P*-value	
Age (*n* = 12)	66.42 (9.78)	67.58 (8.89)	0.83	
Gender (M/F)	4/8	4/8	1.00	

	**Pre-ECT mean (SD)**	**Post-ECT mean (SD)**	***P*-value**	**Cohen’s *d***

**HDRS-24**				
Remitters (*n* = 9)	34.56 (10.02)	2.89 (2.93)	<0.01	4.29
Non-remitters (*n* = 3)	33.67 (6.66)	18.33 (3.51)	0.11	2.88
**HES**				
Remitters	13.22 (2.86)	0.67 (0.71)	<0.01	6.02
Non-remitters	11.00 (3.61)	5.33 (3.06)	0.11	1.69
**RBANS (*n* = 10)**				
Total scale	77.10 (22.70)	80.20 (23.22)	0.43	−0.14
Immediate memory	70.54 (25.09)	81.54 (25.48)	0.10	−0.44
Visual spatial/construction	84.50 (24.42)	87.20 (24.56)	0.66	−0.11
Language	90.55 (10.33)	89.64 (17.53)	0.84	0.06
Attention	82.00 (25.13)	80.10 (24.32)	0.57	0.08
Delayed memory	80.80 (23.79)	81.80 (23.39)	0.79	−0.04
**Trails (*n* = 7)**				
Trails A (s)	64.14 (29.19)	50.57 (14.01)	0.11	0.59
Trails B (s)	181.57 (85.95)	148.00 (89.57)	0.26	0.38

*HDRS-24, Hamilton Depression Rating Scale – 24 item; HES, Hamilton Endogenomorphy Scale; RBANS, repeatable battery for neuropsychological status*.

### Clinical assessments

**Figure 1:**
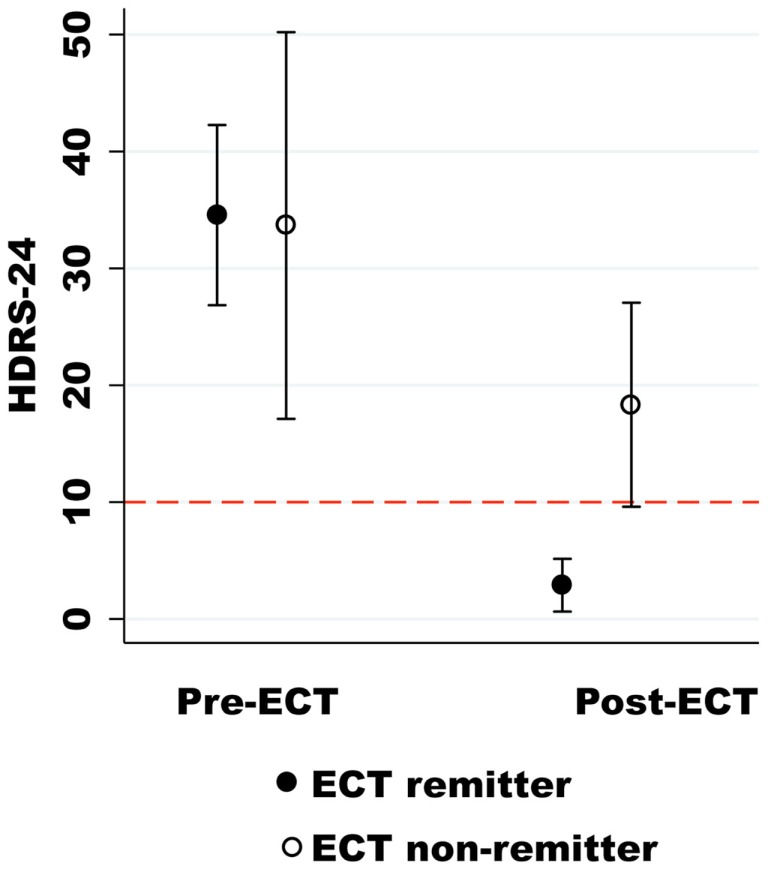
**The Hamilton Depression Rating Scale-24 (HDRS-24) is on the *y*-axis, and the pre- and post-ECT depression ratings are on the *x*-axis**. The dashed red line (HDRS-24) differentiates ECT remitters from non-remitters.

Subjects completed the post-ECT assessment and imaging scan at least 5 days after their last treatment to minimize the effect of the seizure on the imaging results (mean 21.13 ± 13.90 days after the last ECT treatment). The post-ECT HDRS-24 confirmed remission from a pre-ECT assessment of 34.56 ± 10.03 to a post-ECT assessment of 2.89 ± 2.93 post-ECT for nine of the twelve subjects (*z* = 2.67, *P* = 0.0076). The ECT remitters also had a similar reduction in the HES from a pre-ECT assessment of 13.22 ± 2.86 to a post-ECT assessment of 0.67 ± 0.71 subjects (*z* = 2.67, *P* = 0.0074). The average post-ECT HDRS-24 for the non-remitter group also demonstrated a non-significant trend toward clinical improvement from a pre-ECT assessment of 33.67 ± 6.66 to a post-ECT assessment of 18.33 ± 3.51 (*z* = 1.60, *P* = 0.10) as shown in Figure 1. The neuropsychological indices did not show any significant differences before and after ECT (*P* > 0.05).

### Components of interest

**Figure 2:**
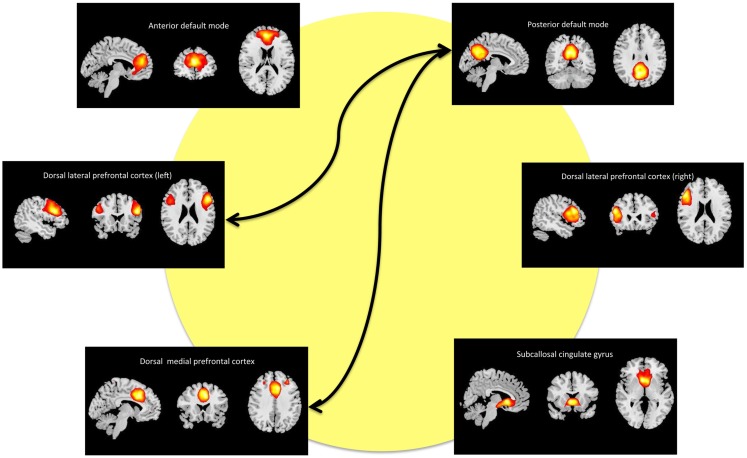
**Maps of the networks of interest include the anterior default mode network (a_DM), subcallosal cingulate gyrus (SCC), dorsal medial prefrontal cortex (DMPFC), posterior default mode network (p_DM), and dorsal lateral prefrontal cortex (r_DLPFC, l_DPLFC)**. Each component map has a lower threshold of *t* = 10. The images are shown in radiological convention. The arrows represent the significant (FDR-corrected), longitudinal differences in FNC associated with ECT response.

We refer to the individual components by functional spatial map: anterior default mode (a_DM), SCC, DMPFC, posterior default mode (p_DM), and (right/left) dorsal lateral prefrontal cortex (r_DLPFC, l_DLPFC). Figure 2 displays the selected components of interest and Table 2 details the anatomic locations (Brodmann areas) of the selected components.

**Table 2:** **Networks of interest**.

Area	Brodmann areas	R/L (cm^3^)	R/L (max-*t*, MNI coordinates)
**ANTERIOR DEFAULT MODE (a_DM)**			
Anterior cingulate	10, 24, 32	6.3/6.8	27.3 (−3, 47, 9)/24.2 (3, 44, 12)
Superior frontal gyrus	9, 10	6.8/5.9	24.6 (−3, 54, 28)/18.4 (18, 51, 22)
Middle frontal gyrus	10	0.5/0.7	14.6 (−24, 51, 20)/15.9 (24, 53, 19)
Cingulate gyrus	32	0.2/0.2	13.6 (−3, 36, 26)/14.5 (3, 36, 26)
Subcallosal gyrus	25	0.0/0.1	(_, _, _)/12.2 (3, 23, −11)
**SUBCALLOSAL CINGULATE GYRUS (SCC)**			
Caudate		1.3/1.2	31.4 (−6, 14, −3)/27.4 (6, 11, −3)
Anterior cingulate	10, 24, 25, 32	3.4/3.8	26.8 (−3, 11, −3)/25.3 (3, 14, −6)
Medial frontal gyrus	10, 11	0.6/0.8	17.0 (−9, 26, −11)/23.0 (6, 26, −11)
Subcallosal gyrus	11, 13, 25, 47	1.2/1.0	21.0 (−3, 14, −11)/21.6 (3, 20, −11)
Lentiform nucleus		0.4/0.2	14.1 (−12, 6, −5)/14.1 (12, 6, −3)
Inferior frontal gyrus	47	0.1/0.2	10.3 (−21, 14, −16)/12.7 (21, 17, −16)
Parahippocampal gyrus		0.1/0.0	10.1 (−12, −7, −15)/(_, _, _)
**DORSAL MEDIAL PREFRONTAL CORTEX (DMPFC)**			
Cingulate gyrus	24, 32	6.0/6.5	22.1 (−3, 11, 38)/26.0 (6, 22, 32)
Medial frontal gyrus	6, 8, 9, 32	4.1/3.2	20.8 (−3, 23, 43)/21.8 (3, 25, 40)
Middle frontal gyrus	9	0.3/1.0	11.9 (−30, 33, 29)/21.7 (30, 36, 29)
Anterior cingulate	24, 32, 33	2.0/1.9	15.5 (−3, 10, 24)/16.3 (3, 16, 24)
Superior frontal gyrus	6, 8, 9	1.3/1.4	14.3 (−3, 34, 43)/14.7 (33, 37, 31)
Paracentral lobule	5, 31	0.1/0.2	10.5 (0, −24, 43)/11.0 (3, −21, 43)
Postcentral gyrus	5	0.0/0.1	(_, _, _)/10.8 (6, −40, 66)
Insula	13	0.1/0.0	10.1 (−33, 14, −3)/(_, _, _)
**POSTERIOR DEFAULT MODE (p_DM)**			
Precuneus	7, 23, 31	14.3/12.2	29.8 (−3, −57, 30)/28.0 (3, −57, 30)
Cingulate gyrus	23, 31	5.7/3.5	29.2 (−6, −57, 28)/25.5 (6, −54, 28)
Cuneus	7	0.4/0.3	27.9 (−6, −65, 31)/20.8 (6, −65, 31)
Posterior cingulate	23, 29, 30, 31	4.4/3.4	25.7 (−6, −54, 25)/26.3 (9, −54, 25)
**RIGHT DORSAL LATERAL PREFRONTAL CORTEX (r_DLPFC)**			
Inferior frontal gyrus	9, 13, 44, 45, 46, 47	16.5/4.4	27.9 (−50, 27, 18)/18.0 (48, 21, 7)
Middle frontal gyrus	9, 10, 46, 47	5.8/0.1	24.3 (−48, 27, 21)/10.9 (48, 33, 15)
Insula	13	4.9/0.2	22.9 (−45, 12, 2)/10.9 (39, 15, 10)
Precentral gyrus	6, 44	3.1/0.3	22.4 (−50, 9, 13)/18.1 (50, 18, 7)
Superior temporal gyrus	22	0.8/0.0	17.0 (−45, 11, −3)/(_, _, _)
Inferior temporal gyrus		0.1/0.0	10.1 (−50, −59, −7)/(_, _, _)
**LEFT DORSAL LATERAL PREFRONTAL CORTEX (l_DLPFC)**			
Inferior frontal gyrus	6, 9, 44, 45, 46	3.9/8.1	18.0 (−48, 10, 27)/30.6 (48, 21, 21)
Middle frontal gyrus	6, 8, 9, 46	6.8/16.1	18.3 (−45, 13, 27)/26.5 (50, 16, 27)
Precentral gyrus	6, 9	0.4/2.9	12.5 (−45, 19, 35)/18.3 (42, 16, 35)
Superior frontal gyrus	8, 9	0.0/0.4	(_, _, _)/11.4 (33, 11, 52)
Inferior parietal lobule	40	0.0/0.1	(_, _, _)/10.4 (53, −46, 22)
Postcentral gyrus		0.0/0.1	(_, _, _)/10.2 (45, −10, 25)

*We applied a one-sample *t*-test to determine the areas of strongest weighting within each ICA spatial map. The anatomic areas described here are the same areas in Figure 2 (each component map has a lower threshold of *t* = 10.)*.

### Functional network connectivity

**Figure 3:**
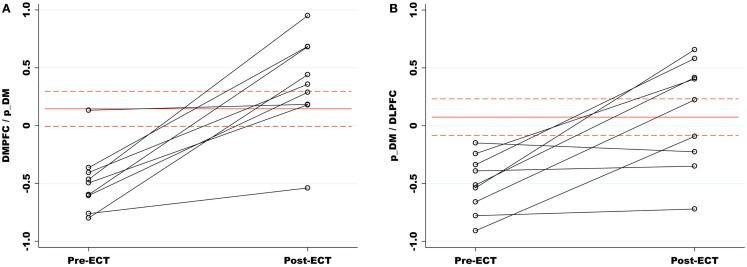
**(A)** Functional network connectivity (FNC) increased between the p_DM and DMPFC pre- to post-ECT. FNC correlations are represented on the *y*-axis. The red lines (dashes) are the means (standard errors) of the matched healthy comparison FNC correlations. Relative to healthy comparisons, the pre-ECT FNC was significantly reduced and normalized with ECT response. **(B)** The p_DM and l_DLPFC had a similar increase in FNC associated with the ECT series. (DMPFC, dorsal medial prefrontal cortex; p_DM, posterior default mode network; l_DLPFC, left dorsal lateral prefrontal cortex.)

Our primary analysis assessed pre- and post-ECT longitudinal changes in FNC among ECT remitters (*n* = 9). Among 15 component correlations, two pairs of components had significant FNC changes associated with ECT response (*P*_FDR_ < 0.05). The FNC measures between p_DM and the DMPFC increased from a negative (*r* = −0.49) to a positive correlation (*r* = 0.36) during the ECT series (*t*_8_ = −5.38, *P* < 0.001). The FNC measures between the p_DM and the l_DLPFC correlation also increased from negative (*r* = −0.50) to a weak positive correlation (*r* = 0.010) during the ECT series (*t*_8_ = −3.85, *P* = 0.0049). These longitudinal, between network changes are shown in Figure 3 and reported in Table 3.

**Table 3:** **Functional network connectivity (FNC) results are shown for the paired- and two-sample *t*-tests**.

Component pair	Pre/post *t*_8_ (*P*)	Pre/HC *t*_16_ (*P*)	Post/HC *t*_16_ (*P*)
a_DM/SCC	0.94 (0.38)		
a_DM/DMPFC	1.38 (0.20)		
a_DM/p_DM	−1.95 (0.09)		
a_DM/r_DLPFC	1.01 (0.34)		
a_DM/l_DLPFC	1.73 (0.12)		
SCC/DMPFC	1.28 (0.24)		
SCC/p_DM	−0.15 (0.89)		
SCC/r_DLPFC	1.27 (0.24)		
SCC/l_DLPFC	2.62 (0.03)		
DMPFC/p_DM	−4.66 (0.002)*	−3.22 (0.005)	0.82 (0.42)
DMPFC/r_DLPFC	0.17 (0.87)		
DMPFC/l_DLPFC	0.09 (0.93)		
p_DM/r_DLPFC	−1.91 (0.09)		
p_DM/l_DLPFC	−3.85 (0.005)*	−3.23 (0.005)	0.11 (0.91)
r_DLPFC/l_DLPFC	0.48 (0.07)		

*The paired *t*-tests between the pre- and post-ECT data within ECT remitters are presented in the second column. *Post hoc* two-sample *t*-tests (third and fourth columns) for the significant pre- and post-ECT component pairs were conducted between the ECT subjects and the healthy comparisons (HC)*.

*False discovery rate significant at *P* < 0.05.

*a_DM, anterior default mode; p_DM, posterior default mode; r_DLPFC, right dorsal lateral prefrontal cortex; l_DLPFC, left dorsal lateral prefrontal cortex; DMPFC, dorsal medial prefrontal cortex; SCC, subcallosal cingulate*.

The secondary analyses focused on the two network pairs that demonstrated significant longitudinal differences after the ECT series. Relative to the healthy subjects, the pre-ECT subjects had significantly lower FNC measures between p_DM and DMPFC (*t*_16_ = −3.22, *P* = 0.005) and the pDMN and l_DLPFC (*t*_16_ = −3.23, *P* = 0.005). The post-ECT and healthy comparison contrasts for both network pairs were not significant (*P* > 0.05). Pairwise correlations between changes in FNC and symptom changes were not significant, whether performed over all 12 ECT subjects or the 9 remitters only (*P* > 0.05).

The two-factor ANOVA comparing groups (ECT remitters and non-remitters) and time (pre- and post-ECT) had significant group × time interactions for p_DM and l_DLPFC (*f*_1, 20_ = 7.52, *P* = 0.013). The FNC measures increased from pre- to post-ECT for the remitters but not for the non-remitters. The interaction was not present with for p_DM and DMPFC (*P* > 0.05). The two-factor ANOVA comparing stimulus delivery (bitemporal and right unilateral) and time (pre- and pot-ECT) was not significant for the stimulus delivery × time interaction for both FNC correlations (*P* > 0.05).

## Discussion

This investigation assessed changes in FNC associated with ECT response in MDD. ECT response reverses the relationship from negative to positive between two pairs of networks: the p_DM/DMPFC and the p_DM/l_DLPFC. Relative to healthy comparisons, both of the aberrant network pairs (i.e., different pre-ECT relative to HC) normalized with ECT response. Although the change in FNC did not predict symptom improvement, the correlation between the p_DM/l_DLPFC did not increase in the ECT non-remitters. The differences between ECT remitters and non-remitters suggest that changes in FNC are related to the therapeutic underpinnings of ECT, as opposed to epiphenomenon.

In order to contextualize our findings with Perrin et al.’s longitudinal resting state fMRI ECT investigation, we compare FNC with seed-voxel correlations. Sheline et al.’s (2010) “hyperconnectivity” hypothesis posits that treatment response during a depressed episode may be associated with reduced seed-voxel functional connectivity, which is supported by the Perrin et al. (2012). Perrin et al.’s (2012) investigation demonstrated reduced functional connectivity with seed-voxel correlations in the l_DLPFC. Their analysis, as they point out, did not indicate a change in connectivity between the DLPFC and specific other regions, but that the overall connectivity from the DLPFC to the rest of the brain was changed with ECT treatment. Seed-voxel correlations are the summation of ICA-derived within network connectivities (power) and ICA-derived between network connectivities (FNC; Joel et al., 2011). Thus, FNC is a part of the seed-voxel functional connectivity totality. Our results, which show increased temporal coherence between anterior and posterior independent components with ECT response (i.e., increased FNC) offer more specificity regarding type and direction change between components associated with recovery from a depressed episode.

Similar to Perrin’s investigation, ECT response was specific for the l_DLPFC despite the different analysis methods. Previous cross-sectional investigations have established the relationship between depression severity and cognitive deficits with aberrant connectivity between the dorsal lateral prefrontal and default mode regions (Vasic et al., 2009; Goveas et al., 2011). Executive function, largely dependent on intact prefrontal and frontal lobe performance, has emerged as one of the core cognitive deficits in major depression and may be related to deficits in attentional control and maladaptive ruminative thought (Austin et al., 2001).

Electroconvulsive therapy response was associated with increased FNC (or loss of anticorrelation) between the dorsal lateral prefrontal cortex and the default mode. We offer two potential explanations regarding the loss of the well-established anticorrelation between these two regions (Fox et al., 2005). First, our sample is older and age-related changes, which show diminished anticorrelations between these networks, may provide the context for interpreting the direction of change from negative to weakly positive (Wu et al., 2011). Second, dynamic FNC changes, as opposed to the implicit assumption of “stationarity” (i.e., the relationship between components does not change during the fMRI run), may also explain the increased FNC correlations (Allen et al., 2012). In healthy participants, the default mode and dorsal attentional systems have been established as “zones of instability” characterized by functional connections that “emerge and dissolve” (Allen et al., 2012). Although not tested in this investigation, ECT response may normalize dynamic FNC among the zones of instability resulting in the return of the ebb and flow of negative and positive relationships between these networks.

Electroconvulsive therapy response may have anatomic specificity with respect to FNC differences. The p_DM network, which has been implicated in depression conceivably through its role in maladaptive, depressive ruminations (Hamilton et al., 2011), is involved in both of the between network changes and appears to be a FNC “hub” for network changes in the context of ECT response. In contrast, the SCC, which has been the target of therapeutic interventions from antidepressant medications to deep brain stimulation, is not involved with any between network changes tested in this investigation, despite being extensively implicated in the pathophysiology of MDD. Normally, therapeutic interventions in this area are associated with reduced activity (Hamani et al., 2011). In the absence of between network changes, our data suggests that the SCC may be more impacted by within network changes in the context of ECT response. Larger investigations are needed to confirm these findings.

Some limitations of this investigation should be acknowledged. First, the small sample size limited further analyses between clinical and treatment variables (e.g., psychotic versus non-psychotic depression). Second, MDD subjects were medicated at both assessment points, confounding a straightforward interpretation of ECT effects. Because of the acuity of the depressed episodes, withdrawing medications would not have been feasible prior to the first imaging assessment, and the expert consensus is that antidepressant medications act synergistically with ECT to enhance response (Weiner et al., 2001; Sackeim et al., 2009). However, only 3 of the 12 subjects had modification in their antidepressant drug therapy during the ECT course, reducing the likelihood of a confounding effect of the medications. However, antidepressant medications may also reduce functional connectivity (McCabe and Mishor, 2011). As previously discussed, FNC is a part of the seed-voxel functional connectivity totality (Joel et al., 2011) and FNC is also measuring functional connectivity (Joel et al., 2011). We hope to study these effects in future work with more extensive numbers of subjects on different medication levels.

In conclusion, this research enhances our understanding of the functional neural correlates of ECT response. Continued research in this area may differentiate ECT remitters from non-remitters prior to the ECT series and identify patients at risk of relapse immediately following the ECT series, an essential step in the development of biomarkers for treatment response in MDD. Results from this study may also be applicable to a spectrum of treatments for MDD of varying invasivity. For example, many focal neuromodulation treatments have excellent safety profiles, such as transcranial magnetic stimulation or transcranial direct-current stimulation, which do not require anesthesia and have the potential for widespread use beyond academic medical centers and large, metropolitan hospitals (Pascual-Leone et al., 1996). Despite this safety profile, the speed of response and efficacy of other neural modulation treatments does not match the “gold-standard” of ECT (Eranti et al., 2007; Kalu et al., 2012). A better understanding of ECT response may improve the efficacy of potentially safer, more accessible treatments for MDD.

## Conflict of Interest Statement

Dr. Vince D. Calhoun has received research support from the National Institutes of Health, National Science Foundation, Department of Energy; has done some legal consultation; has performed grant reviews for the National Institutes of Health and other agencies; has guest-edited journal sections; has given academic lectures in various scientific venues; has received support for various training courses; and has generated books or book chapters for publishers of various texts. None of the other authors report conflicts of interest.

## Footnotes

^1^http://www.fil.ion.ucl.ac.uk/spm/software/spm5

^2^http://mialab.mrn.org/software/gift

^3^http://mialab.mrn.org/software/fnc

## References

[B1] Allen E. A. Damaraju E. Plis S. M. Erhardt E. B. Eichele T. Calhoun V. D. (2012). Tracking whole-brain connectivity dynamics in the resting state. Cereb. Cortex. 10.1093/cercor/bhs352 PMC3920766 23146964

[B2] Allen E. A. Erhardt E. B. Damaraju E. Gruner W. Segall J. M. Silva R. F. (2011). A baseline for the multivariate comparison of resting-state networks. Front. Syst. Neurosci. 5:2 10.3389/fnsys.2011.00002 21442040 PMC3051178

[B3] Austin M. P. Mitchell P. Goodwin G. M. (2001). Cognitive deficits in depression: possible implications for functional neuropathology. Br. J. Psychiatry 178, 200–206 10.1192/bjp.178.3.200 11230029

[B4] Beall E. B. Malone D. A. Dale R. M. Muzina D. J. Koenig K. A. Bhattacharrya P. K. (2012). Effects of electroconvulsive therapy on brain functional activation and connectivity in depression. J. ECT 28, 234–241 22820953 10.1097/YCT.0b013e31825ebcc7

[B5] Biswal B. Yetkin F. Z. Haughton V. M. Hyde J. S. (1995). Functional connectivity in the motor cortex of resting human brain using echo-planar MRI. Magn. Reson. Med. 34, 537–541 10.1002/mrm.1910340409 8524021

[B6] Calhoun V. D. Adali T. (2012). Multi-subject independent component analysis of fMRI: a decade of intrinsic networks, default mode, and neurodiagnostic discovery. IEEE Rev. Biomed. Eng. 5, 60–73 10.1109/RBME.2012.2211076 23231989 PMC4433055

[B7] Calhoun V. D. Adali T. Pearlson G. D. Pekar J. J. (2001). A method for making group inferences from functional MRI data using independent component analysis. Hum. Brain Mapp. 14, 140–151 10.1002/hbm.1048 11559959 PMC6871952

[B8] Calhoun V. D. Kiehl K. A. Pearlson G. D. (2008). Modulation of temporally coherent brain networks estimated using ICA at rest and during cognitive tasks. Hum. Brain Mapp. 29, 828–838 10.1002/hbm.20463 18438867 PMC2649823

[B9] Cordes D. Haughton V. M. Arfanakis K. Wendt G. J. Turski P. A. Moritz C. H. (2000). Mapping functionally related regions of brain with functional connectivity MR imaging. AJNR Am. J. Neuroradiol. 21, 1636–1644 11039342 PMC8174861

[B10] Eranti S. Mogg A. Pluck G. Landau S. Purvis R. Brown R. G. (2007). A randomized, controlled trial with 6-month follow-up of repetitive transcranial magnetic stimulation and electroconvulsive therapy for severe depression. Am. J. Psychiatry 164, 73–81 10.1176/appi.ajp.164.1.73 17202547

[B11] Erhardt E. B. Allen E. A. Damaraju E. Calhoun V. D. (2011a). On network derivation, classification, and visualization: a response to Habeck and Moeller. Brain Connect. 1, 1–19 10.1089/brain.2011.0022 21808745 PMC3146759

[B12] Erhardt E. B. Rachakonda S. Bedrick E. J. Allen E. A. Adali T. Calhoun V. D. (2011b). Comparison of multi-subject ICA methods for analysis of fMRI data. Hum. Brain Mapp. 32, 2075–2095 10.1002/hbm.21170 21162045 PMC3117074

[B13] Filippi M. Agosta F. Scola E. Canu E. Magnani G. Marcone A. (2012). Functional network connectivity in the behavioral variant of frontotemporal dementia. Cortex. 10.1016/j.cortex.2012.09.017 23164495

[B14] Fox M. D. Snyder A. Z. Vincent J. L. Corbetta M. Van Essen D. C. Raichle M. E. (2005). The human brain is intrinsically organized into dynamic, anticorrelated functional networks. Proc. Natl. Acad. Sci. U.S.A. 102, 9673–9678 10.1073/pnas.0409470102 15976020 PMC1157105

[B15] Freire L. Roche A. Mangin J. F. (2002). What is the best similarity measure for motion correction in fMRI time series? IEEE Trans. Med. Imaging 21, 470–484 10.1109/TMI.2002.1009383 12071618

[B16] Genovese C. R. Lazar N. A. Nichols T. (2002). Thresholding of statistical maps in functional neuroimaging using the false discovery rate. Neuroimage 15, 870–878 10.1006/nimg.2001.1037 11906227

[B17] Goveas J. Xie C. Wu Z. Douglas Ward B. Li W. Franczak M. B. (2011). Neural correlates of the interactive relationship between memory deficits and depressive symptoms in nondemented elderly: resting fMRI study. Behav. Brain Res. 219, 205–212 10.1016/j.bbr.2011.01.008 21238490 PMC3062733

[B18] Greicius M. D. Flores B. H. Menon V. Glover G. H. Solvason H. B. Kenna H. (2007). Resting-state functional connectivity in major depression: abnormally increased contributions from subgenual cingulate cortex and thalamus. Biol. Psychiatry 62, 429–437 10.1016/j.biopsych.2006.09.020 17210143 PMC2001244

[B19] Guo C. C. Kurth F. Zhou J. Mayer E. A. Eickhoff S. B. Kramer J. H. (2012). One-year test-retest reliability of intrinsic connectivity network fMRI in older adults. Neuroimage 61, 1471–1483 10.1016/j.neuroimage.2012.03.027 22446491 PMC4226138

[B20] Hamani C. Mayberg H. Stone S. Laxton A. Haber S. Lozano A. M. (2011). The subcallosal cingulate gyrus in the context of major depression. Biol. Psychiatry 69, 301–308 10.1016/j.biopsych.2010.10.012 21145043

[B21] Hamilton J. P. Furman D. J. Chang C. Thomason M. E. Dennis E. Gotlib I. H. (2011). Default-mode and task-positive network activity in major depressive disorder: implications for adaptive and maladaptive rumination. Biol. Psychiatry 70, 327–333 10.1016/j.biopsych.2011.02.003 21459364 PMC3144981

[B22] Harrison G. Hopper K. Craig T. Laska E. Siegel C. Wanderling J. (2001). Recovery from psychotic illness: a 15- and 25-year international follow-up study. Br. J. Psychiatry 178, 506–517 10.1192/bjp.178.6.506 11388966

[B23] Hermann R. C. Dorwart R. A. Hoover C. W. Brody J. (1995). Variation in ECT use in the United States. Am. J. Psychiatry 152, 869–875 7755116 10.1176/ajp.152.6.869

[B24] Jafri M. J. Pearlson G. D. Stevens M. Calhoun V. D. (2008). A method for functional network connectivity among spatially independent resting-state components in schizophrenia. Neuroimage 39, 1666–1681 10.1016/j.neuroimage.2007.11.001 18082428 PMC3164840

[B25] Joel S. E. Caffo B. S. Van Zijl P. C. Pekar J. J. (2011). On the relationship between seed-based and ICA-based measures of functional connectivity. Magn. Reson. Med. 66, 644–657 10.1002/mrm.22818 21394769 PMC3130118

[B26] Kalu U. G. Sexton C. E. Loo C. K. Ebmeier K. P. (2012). Transcranial direct current stimulation in the treatment of major depression: a meta-analysis. Psychol. Med. 42, 1791–1800 10.1017/S0033291711003059 22236735

[B27] Kellner C. H. Knapp R. Husain M. M. Rasmussen K. Sampson S. Cullum M. (2010). Bifrontal, bitemporal and right unilateral electrode placement in ECT: randomised trial. Br. J. Psychiatry 196, 226–234 10.1192/bjp.bp.109.066183 20194546 PMC2830057

[B28] Kellner C. H. Knapp R. G. Petrides G. Rummans T. A. Husain M. M. Rasmussen K. (2006). Continuation electroconvulsive therapy vs pharmacotherapy for relapse prevention in major depression: a multisite study from the Consortium for Research in Electroconvulsive Therapy (CORE). Arch. Gen. Psychiatry 63, 1337–1344 10.1001/archpsyc.63.12.1337 17146008 PMC3708140

[B29] Koch W. Teipel S. Mueller S. Buerger K. Bokde A. L. Hampel H. (2010). Effects of aging on default mode network activity in resting state fMRI: does the method of analysis matter? Neuroimage 51, 280–287 10.1016/j.neuroimage.2009.12.008 20004726

[B30] Kochiyama T. Morita T. Okada T. Yonekura Y. Matsumura M. Sadato N. (2005). Removing the effects of task-related motion using independent-component analysis. Neuroimage 25, 802–814 10.1016/j.neuroimage.2004.12.027 15808981

[B31] Lui S. Li T. Deng W. Jiang L. Wu Q. Tang H. (2010). Short-term effects of antipsychotic treatment on cerebral function in drug-naive first-episode schizophrenia revealed by “resting state” functional magnetic resonance imaging. Arch. Gen. Psychiatry 67, 783–792 10.1001/archgenpsychiatry.2010.84 20679586

[B32] Mayberg H. S. (2003). Modulating dysfunctional limbic-cortical circuits in depression: towards development of brain-based algorithms for diagnosis and optimised treatment. Br. Med. Bull. 65, 193–207 10.1093/bmb/65.1.193 12697626

[B33] Mayberg H. S. Lozano A. M. Voon V. McNeely H. E. Seminowicz D. Hamani C. (2005). Deep brain stimulation for treatment-resistant depression. Neuron 45, 651–660 10.1016/j.neuron.2005.02.014 15748841

[B34] McCabe C. Mishor Z. (2011). Antidepressant medications reduce subcortical-cortical resting-state functional connectivity in healthy volunteers. Neuroimage 57, 1317–1323 10.1016/j.neuroimage.2011.05.051 21640839 PMC3141109

[B35] McKeown M. J. Hansen L. K. Sejnowsk T. J. (2003). Independent component analysis of functional MRI: what is signal and what is noise? Curr. Opin. Neurobiol. 13, 620–629 10.1016/j.conb.2003.09.012 14630228 PMC2925426

[B36] McKeown M. J. Makeig S. Brown G. G. Jung T. P. Kindermann S. S. Bell A. J. (1998). Analysis of fMRI data by blind separation into independent spatial components. Hum. Brain Mapp. 6, 160–188 10.1002/(SICI)1097-0193(1998)6:3<160::AID-HBM5>3.3.CO;2-R 9673671 PMC6873377

[B37] Pascual-Leone A. Rubio B. Pallardo F. Catala M. D. (1996). Rapid-rate transcranial magnetic stimulation of left dorsolateral prefrontal cortex in drug-resistant depression. Lancet 348, 233–237 10.1016/S0140-6736(96)01219-6 8684201

[B38] Perrin J. S. Merz S. Bennett D. M. Currie J. Steele D. J. Reid I. C. (2012). Electroconvulsive therapy reduces frontal cortical connectivity in severe depressive disorder. Proc. Natl. Acad. Sci. U.S.A. 109, 5464–5468 10.1073/pnas.1117206109 22431642 PMC3325678

[B39] Randolph C. Tierney M. C. Mohr E. Chase T. N. (1998). The Repeatable Battery for the Assessment of Neuropsychological Status (RBANS): preliminary clinical validity. J. Clin. Exp. Neuropsychol. 20, 310–319 10.1076/jcen.20.3.310.823 9845158

[B40] Reitan R. M. (1958). Validity of the trail making test as an indicator of organic brain damage. Percept. Mot. Skills 8, 271–276 10.2466/PMS.8.7.271-276

[B41] Sackeim H. A. Dillingham E. M. Prudic J. Cooper T. McCall W. V. Rosenquist P. (2009). Effect of concomitant pharmacotherapy on electroconvulsive therapy outcomes: short-term efficacy and adverse effects. Arch. Gen. Psychiatry 66, 729–737 10.1001/archgenpsychiatry.2009.75 19581564

[B42] Sackeim H. A. Haskett R. F. Mulsant B. H. Thase M. E. Mann J. J. Pettinati H. M. (2001). Continuation pharmacotherapy in the prevention of relapse following electroconvulsive therapy: a randomized controlled trial. JAMA 285, 1299–1307 10.1001/jama.285.10.1299 11255384

[B43] Schmidt E. Z. Reininghaus B. Enzinger C. Ebner C. Hofmann P. Kapfhammer H. P. (2008). Changes in brain metabolism after ECT-positron emission tomography in the assessment of changes in glucose metabolism subsequent to electroconvulsive therapy–lessons, limitations and future applications. J. Affect. Disord. 106, 203–208 10.1016/j.jad.2007.06.009 17662472

[B44] Scott A. Courtney W. Wood D. De La Garza R. Lane S. King M. (2011). COINS: an innovative informatics and neuroimaging tool suite built for large heterogeneous datasets. Front. Neuroinform. 5:33 10.3389/fninf.2011.00033 22275896 PMC3250631

[B45] Shehzad Z. Kelly A. M. Reiss P. T. Gee D. G. Gotimer K. Uddin L. Q. (2009). The resting brain: unconstrained yet reliable. Cereb. Cortex 19, 2209–2229 10.1093/cercor/bhn256 19221144 PMC3896030

[B46] Sheline Y. I. Price J. L. Yan Z. Mintun M. A. (2010). Resting-state functional MRI in depression unmasks increased connectivity between networks via the dorsal nexus. Proc. Natl. Acad. Sci. U.S.A. 107, 11020–11025 10.1073/pnas.1000446107 20534464 PMC2890754

[B47] Thase M. E. Hersen M. Bellack A. S. Himmelhoch J. M. Kupfer D. J. (1983). Validation of a Hamilton subscale for endogenomorphic depression. J. Affect. Disord. 5, 267–278 10.1016/0165-0327(83)90050-2 6224838

[B48] Vasic N. Walter H. Sambataro F. Wolf R. C. (2009). Aberrant functional connectivity of dorsolateral prefrontal and cingulate networks in patients with major depression during working memory processing. Psychol. Med. 39, 977–987 10.1017/S0033291708004443 18845009

[B49] Weiner R. D. Coffey C. E. Fochtmann L. J. Greenberg R. M. Isenberg K. E. Kellner C. H. (2001). The Practice of Electroconvulsive Therapy: Recommendations for Treatment, Training, and Privileging. Washington, DC: American Psychiatric Association

[B50] Wu J. T. Wu H. Z. Yan C. G. Chen W. X. Zhang H. Y. He Y. (2011). Aging-related changes in the default mode network and its anti-correlated networks: a resting-state fMRI study. Neurosci. Lett. 504, 62–67 10.1016/j.neulet.2011.08.059 21925236

[B51] Ystad M. Eichele T. Lundervold A. J. Lundervold A. (2010). Subcortical functional connectivity and verbal episodic memory in healthy elderly – a resting state fMRI study. Neuroimage 52, 379–388 10.1016/j.neuroimage.2010.03.062 20350608

